# Retraction Note: Rifaximin alters gut microbiota profile, but does not affect systemic inflammation - a randomized controlled trial in common variable immunodeficiency

**DOI:** 10.1038/s41598-024-54117-6

**Published:** 2024-02-14

**Authors:** S. F. Jørgensen, M. E. Macpherson, T. Bjørnetrø, K. Holm, M. Kummen, A. Rashidi, A. E. Michelsen, T. Lekva, B. Halvorsen, M. Trøseid, T. E. Mollnes, R. K. Berge, A. Yndestad, T. Ueland, T. H. Karlsen, P. Aukrust, J. R. Hov, B. Fevang

**Affiliations:** 1 Research Institute of Internal Medicine, Division of Surgery, Inflammatory Diseases and Transplantation, Oslo University Hospital, Rikshospitalet, Norway; 2 Section of Clinical Immunology and Infectious Diseases, Department of Rheumatology, Dermatology and Infectious Diseases, Oslo University Hospital, Rikshospitalet, Norway; 3 Institute of Clinical Medicine, University of Oslo, Oslo, Norway; 4 Norwegian PSC Research Center, Division of Surgery, Inflammatory Diseases and Transplantation, Oslo University Hospital Rikshospitalet, Oslo, Norway; 5 Research Laboratory, Nordland Hospital, Bodø, and Faculty of Health Sciences, K.G. Jebsen TREC, University of Tromsø, Tromsø, Norway; 6 Centre of Molecular Inflammation Research, Norwegian University of Science and Technology, Trondheim, Norway; 7 Department of Immunology, Oslo University Hospital, and University of Oslo, Oslo, Norway; 8 Department of Clinical Science, University of Bergen, Bergen, Norway; 9 Department of Heart Disease, Haukeland University Hospital, Bergen, Norway

Retraction of: *Scientific Reports* 10.1038/s41598-018-35367-7, published online 17 January 2019

The Authors have retracted this Article.

Recent probiotic use was an exclusion criterion for participation in the randomised clinical trial (RCT) reported in the Article. Following publication, the authors identified via dietary data collected concomitantly, but outside the study protocol, that 26% of the trial participants may have been using probiotics despite reporting no use at the time of inclusion. While there had been no deliberate violation of the protocol, if these participants are retrospectively excluded the statistical power of the RCT falls below 80%.

All authors agree to this retraction.

